# Is Antimicrobial Resistance a Slowly Emerging Disaster?

**DOI:** 10.1093/phe/phv015

**Published:** 2015-06-30

**Authors:** A. M. Viens, Jasper Littmann

**Affiliations:** Southampton Law School, University of Southampton; Institute of Experimental Medicine, Christian-Albrechts University Kiel

## Abstract

The problem of antimicrobial resistance is so dire that people are predicting that the era of antibiotics may be coming to an end, ushering in a ‘post-antibiotic’ era. A comprehensive policy response is therefore urgently needed. A part of this response will require framing the problem in such a way that adequately reflects its nature as well as encompassing an approach that has the best prospect of success. This paper considers framing the problem as a slowly emerging disaster, including its potential benefits and difficulties, from a conceptual and policy perspective.

On 7 April 2011, World Health Day, the World Health Organization (WHO) announced that urgent actions were necessary if the effectiveness of antibiotics was to be ensured in the future. Failure to confront the rising problem of antimicrobial resistance (AMR) would result in the loss of the ‘miracle cures’ offered by antibiotics, the WHO’s Director-General Margaret Chan announced (WHO, 2011).^1^ These worries have been reiterated by Dame Sally Davies, the Chief Medical Officer for England, who described AMR as both a ‘catastrophic threat’ and an ‘apocalyptical threat’ (McCarthy, 2013; Sample, 2013). She also ensured that AMR was addressed within the national risk register—the government’s assessment of the likelihood and potential impact of civil emergency risks—together with national threats such as major flooding, terrorist attacks and pandemic outbreaks (Cabinet Office, 2015: 15–16).^2^ In a recent publication, Davies and her colleagues (2013) warned of the possibility that health care systems might no longer be able to treat bacterial diseases effectively in as little as 20 years. Similar concerns have been voiced by others, for instance, by The World Economic Forum (2013: 28), which declared last year that AMR constitutes one of the main risks to human health.

AMR is not, however, merely a future threat—the present human and economic costs of AMR already amount to thousands of casualties each year, increase the infectious disease burden around the world and leads to billions of dollars in direct health costs and lost productivity (CDC, 2013; Smith and Coast, 2013; Review on Antimicrobial Resistance, 2014). What exacerbates the problem is that there is no realistic chance of a timely medical or technological solution to the problem—there are currently few new antibiotics under development and those that lack novel action mechanisms that could effectively circumvent existing AMR (Mossialos *et al.*, 2010; Cars and Nathan, 2014). Moreover, AMR is a distinctly global problem—due to international travel and exchange of goods, bacterial infections cannot be contained within national boundaries.

The problem of AMR is so dire that people are predicting that the era of antibiotics may be coming to an end, ushering in a ‘post-antibiotic’ era in which we will be as vulnerable to bacterial infections as we once were before Fleming’s discovery (Brown, 1994). According to the WHO (2014: ix), ‘the problem is so serious that it threatens the achievements of modern medicine. A post-antibiotic era—in which common infections and minor injuries can kill—is a very real possibility for the 21st century’. Returning to a time without effective antibiotics would have disastrous consequences over life spans and across generations. A comprehensive policy response is therefore urgently needed. A part of this response will require framing the problem in such a way that it adequately reflects its nature as well as encompassing an approach that has the best prospect of success.

The aim of this article is two fold. We begin, firstly, by exploring whether it is plausible, at least *prima facie*, to conceptualize AMR as a type of disaster; in particular, as a slowly emerging disaster. We examine some reasons in favor of doing so, as well as some tensions and problems involved in such a conceptualization. In examining the conceptual question of how we should go about understanding which circumstances deserve the label of disaster, we seek to highlight that events or processes, such as AMR, are notably different from what are thought of as ‘typical disasters’ and how this might require us to think more deeply about how we should go about defining disasters, as well as why we attach the label of disaster to particular circumstances. Whether or not we have overwhelming reason to include AMR within the category of disaster, we may still have pragmatic reasons for approaching the problem of AMR as if it is some kind of disaster. We then proceed, secondly, to outline different policy-based considerations that might give us reason to approach AMR as if it is a disaster. These conceptual and policy considerations should be analyzed separately, even though there are many connections between them. It is very important that the question of ‘what is a disaster?’ should be addressed independently from the question of ‘how should we respond to a disaster?’, even though the former will bear on the latter without necessarily determining it. Nevertheless, it is still fruitful to investigate how framing certain problems as disasters can shape our approaches to them in a way that allows practitioners and policy makers to import existing knowledge and infrastructure to AMR, which will be our primary concern.

## AMR: Challenge, Threat or Disaster?

It is indisputable that AMR is a major threat to individual and public health. In recent years, much progress has been made in raising awareness for this fact and the problem is widely recognized by clinicians, scientists, national governments and global health agencies (Laxminarayan *et al.*, 2013; Davies *et al.*, 2013; Nature, 2013; President's Council of Advisors on Science and Technology, 2014; World Health Organization, 2014). It is also starting to get further attention by philosophers and lawyers examining the many normative implications of AMR (Selgelid, 2007; Anomaly, 2009, 2010; Millar, 2012; Hoffman *et al.*, 2015). As this recognition has increased, so have the numerous challenges it will present for patients, clinicians, public health officials, policy makers, as well as the pharmaceutical and agricultural industries. In light of AMR’s severity and the risk it poses to public health, it appears intuitively plausible to describe it as a disaster. What we wish to explore further in this article is whether or not such a categorization is warranted.

Unfortunately, the label of disaster—or even terms such as epidemic and pandemic—are often arbitrarily attached to large-scale health problems in an effort for them to receive more governmental funding, policy prioritization, media attention or public awareness. For instance, even among clinicians, scientists, politicians and journalists, problems as varied as vision care (Walker, 2009), autism (Dawson, 2012), opiate abuse (MacQuarrie, 2014), synthetic marijuana (Breiner, 2015), youth unemployment (Marmot, 2011), food poverty (Taylor-Robinson *et al.*, 2013) and obesity (Long, 2012; Callahan, 2013) have been described as public health disasters or epidemics/pandemics. Whether terms such as disaster or pandemic are contested concepts or reflect different health issue prioritizations, how we define and use technical terms matters. We should be very wary of people who throw such terms around so capriciously as synonyms for public health problems that are important, urgent or serious. Nevertheless, given the nature of the problem of AMR, it seems *prima facie* plausible that it might qualify as a disaster. How does it comport with our current ways of defining what constitutes a disaster?

Different jurisdictions define what constitutes a disaster or emergency differently.^3^ Consider a selection of example definitions within national laws and international policy:

a sudden, calamitous event that causes serious disruption to the functioning of a community or a society causing widespread human, material, economic and/or environmental losses which exceed the ability of the affected community or society to cope using its own level of resources (United Nations International Strategy for Disaster Reduction, 2004: 43).

an urgent and critical situation of a temporary nature that (i) seriously endangers the lives, health or safety of Canadians and is of such proportions or nature as to exceed the capacity or authority of a province to deal with it, or (ii) seriously threatens the ability of the Government of Canada to preserve the sovereignty, security and territorial integrity of Canada and that cannot be effectively dealt with under any other law of Canada (Canada, 1985: §3).

(i) an event or situation which threatens serious damage to human welfare in a place in the United Kingdom, (ii) an event or situation which threatens serious damage to the environment of a place in the United Kingdom, or (iii) war, or terrorism, which threatens serious damage to the security of the United Kingdom (United Kingdom, 2004: §1(1)).

While jurisdictions end up defining disasters differently—both in terms of how expansive the definition is and who decides on the classification—there is a high level of congruence among them in terms of how they would label similar events as being disasters. Notice, however, that within these definitions, there is a presumption that disasters are the kinds of circumstances that have a clear temporal boundary as one of its situational features. This is to say, disasters are normally assumed to last only for a particular duration of time. Whether referring to disasters as a distinct circumstance, or using terms such as ‘temporary’ or ‘sudden’, the general presumption is that—in distinguishing disasters as non-normal circumstances—there is some point at which a disaster begins and ends. A disaster is thus an event that comes into and out of existence, and there will be, at least in principle, identifiable periods where we can prepare, respond and recover from such events.^4^ The marks of a disaster are such that they emerge and depart—there has to be an ebb and flow from normal to non-normal back to normal circumstances, even if the periods of transition from these circumstances after a disaster can take days, weeks or months. An example that fits this common sense understanding of what constitutes a disaster is major flooding. There is a normal level for waterways and water bodies that rise to a non-normal level, which exceeds the usual management capabilities and submerges land that is usually dry, before eventually returning back to normal water levels for the region. In distinguishing between times of normalcy and disaster, this characteristic feature of disaster is understood in terms of statistical frequency or predominance within a population or jurisdiction in which they come into and out of existence.^5^

The claim that disasters are temporally bound in this way, however, can be differentiated into two claims—one stronger than the other. We believe there are good reasons for maintaining such claims in our understanding of the nature of disasters.

On the one hand, there is the weaker claim that a disaster has to have some determinant temporal boundary. As such, it is possible to differentiate between times of normalcy and times of disaster and it is possible to identify when these periods begin and end—even if that boundary will often be plagued with problems of vagueness at the borderline. Again, returning to the example of flooding, even when water levels have returned to typically levels for the region, much of the surrounding waterlogged areas will still suffer from population displacement, loss of shelter and electricity, compromised safe water and sanitation, and the increased risk of vector-borne diseases (Keim, 2010). It will often be difficult to precisely say when a major flooding disaster has ceased to exist. This difficulty might be even greater in other such events; for instance, when we should say that the HIV/AIDS epidemic in Sub-Saharan Africa has ended? Nevertheless, in principle, a division between normalcy and disaster indicates there is some temporal boundary even if its determination is complex and difficult to ascertain.

On the other hand, there is the stronger claim that a disaster’s temporal boundary is such that those bounds cannot extend for a duration in which a disaster becomes the norm. According to this stronger claim, it would be impossible to have an indefinite disaster situation. We should resist a notion of disaster that allows temporal boundaries to exist for such an extensive duration that it is possible for communities to live in a permanent disaster. Such an understanding cannot make sense of a disaster as a periodic event that punctuates the experience of normalcy. If a disaster can have a duration of several decades or centuries, then it has become normalcy. Once a disaster becomes the new normal, it would cease to be a disaster as we commonly (and correctly) understand the term.

To be clear, in differentiating the temporal boundaries of a disaster, it need not have any normative implications for how we ought to go about *responding* to such events. For instance, even if the concept of disaster does not currently count AMR as a disaster *per se*, it does not mean AMR does not present an extremely serious threat that government policy and individual action should take measures to mitigate or prevent right now. Concomitantly, it also means that the fact that AMR should be a top priority that requires urgent action need not automatically qualify it as a disaster either. Even if we should endorse the stronger claim that disasters are not the kinds of events that can last for decades or centuries, it must be understood that this need not entail the normative implication that these circumstances are less urgent or less important to respond to. Indeed, this is why it is so important to keep the labeling question separate from the response question—the application of the label of disaster should not be thought to wholly settle the question of whether or to what extent we respond to particular circumstances. The situational features that constitute a disaster will shape both conceptual and normative considerations; however, we must be careful about how we understand and use these considerations in our deliberation and debate about such circumstances.

Part of the reason why some people may be tempted to conceive of disasters as having such massive temporal boundaries is because they are making the mistake of conflating the antecedent conditions that contribute to the instantiation of a disaster as being part of the disaster itself. The two are inextricably linked and extremely important for our understanding of disasters, but they should be kept distinct. We must be careful to distinguish between an event that takes a long time to occur and an event that has a long duration. That is to say, there is a difference between (i) an event *existing* for a long duration and (ii) an event taking a long time to *come into* existence. In the case of the former, a disaster comes into existence, but that circumstance stays in existence for long duration. In the case of the latter, while there will be a number of causal antecedents that could be traced or identified, it would not be until the instantiation of the event itself that a disaster would exist. The notion of a slowly emerging disaster falls into this latter category.

Furthermore, given the complexity of most disasters and the difficulty in predicting their moment of instantiation, there is often no clear, determinate point in time in which factor *X* at time *T* can be pinpointed as the initiation of a disaster. This is certainly the case with AMR. While many microorganisms are becoming resistant to available preventative and therapeutic medicines, it is clear that AMR is not an all-or-nothing phenomenon. Unlike an event such as an earthquake or a terrorist attack, the emergence of AMR is not a distinct, specifiable event that occurs and immediately causes us to go from a time of normalcy to a time of disaster. Moreover, there can be different levels of resistance within a specific class of microbe. For instance, we distinguish between multidrug-resistant tuberculosis and extensively drug-resistant tuberculosis.^6^ It then looks like we would have to have some specific threshold level of resistance at which AMR would become a reality. This further complicates the question how we can determine the beginning of an ‘AMR disaster’.

Moreover, a related concern is that if we retain the claim that what has become normal can no longer be a disaster, then there is a possibility that certain slowly emerging events might never constitute a slowly emerging disaster because each progressive period of time will be viewed as normal until it reaches its end point.^7^ This kind of objection is familiarly raised along the line of the boiling frog analogy. If you were to place a frog in boiling water, it would notice immediately and try to jump out. If you were, however, to place a frog in a pot of room temperature water and very slowly brought it to a boil, the frog would not notice the slow, progressive temperature rise until it was boiled alive. Should we think this analogy, and concomitant worry, holds for AMR? Strictly speaking, it does not hold because it conflates the question of whether being temporally bounded is a situational feature of disaster with the question of whether there can be disasters that take a long time to come into existence. In the case of AMR, if we maintained the temporal boundary criterion of disasters, then we would have to consider it a traditional disaster now and then over time, as the loss of antibiotic effectiveness becomes the new normal, we would then be committed to saying it is no longer a disaster. That, however, is not what is being claimed here. Indeed, the very idea of a slowly emerging disaster is that it presses on this temporal boundary criterion and explores whether it makes sense to retain the idea that disasters punctuate normalcy yet can do so in a way that is slow and progressive. Returning to the analogy, it may be argued, the frog should not care whether it is thrown into boiling water directly or whether it is placed in slowly boiling water—the disaster consists in the fact that it is in boiling water! It is this aspect of the analogy that holds and that speaks to whether AMR should be thought about as a disaster if we are all frogs slowly and obliviously going to end up cooked. The worry is that as time progresses—even if antibiotics are used more prudently, infection control procedures and materials are enhanced, food production and supply chains are improved, more money is put into research for new classes of antibiotics—AMR could still increase over decades until we get to a point where we have reached a post-antibiotic era without thinking at any point it is a disaster. Our contention is that the idea of a slowly emerging disaster may meet such a worry, since it allows us to understand AMR as a type of disaster now yet in a way that would not require us to reach a post-antibiotic era before being able to call it a disaster.^8^

For the purposes of this article, however, we do not need to define the conditions under which we can legitimately conclude that we have entered a post-antibiotic era. We are here merely concerned with the question how we should conceive of such an event. In particular, we are interested in the question of whether it makes sense conceptually to understand widespread AMR and a widespread loss of antibiotic effectiveness as a disaster. While AMR currently need not qualify as a traditional disaster *per se*—that is to say, we are not currently experiencing the disastrous consequences of a post-antibiotic era—it certainly is the kind of event described above as a disaster that takes a long time to come into existence. It is, in this sense, better understood as a slowly emerging disaster, since the antecedent conditions that can lead to a post-antibiotic era are currently in place and are proceeding in such a way that conceivably will lead to what would end up as a commonplace disaster.^9^

As noted earlier, many of the features of AMR can allow it to qualify as a disaster under many of the accepted public policy definitions used. One noted exception is the feature of being temporally bounded. If we waited to fulfill such definitions strictly, we would not be justified in labeling the threat of AMR a disaster at present—only when the post-antibiotic era has been achieved. This would be problematic, as by the time the actual disaster of the post-antibiotic era had been instantiated, the significant risk of harm, loss or burden will have already been actuated and our options to respond will have become constrained and ineffective. The idea of a slowly emerging disaster provides a middle way in thinking about public health threats. It does not attach the label of a full-fledged disaster; instead, it emphasizes the connection between the antecedent conditions and the resulting event—thereby placing the focus on how the antecedent conditions contribute to the disaster taking a long time to come into existence, as well as how this establishes response options to those antecedent conditions.

Another advantage of understanding AMR as a slowly emerging disaster is that it need not completely conform to our commonplace view of full-fledged disasters, in which we go from normalcy to disaster back to normalcy. Our actions to combat AMR will never completely succeed to eliminate all drug resistance. Bacteria will continue to take advantage of the natural and anthropogenic conditions that allow them to mutate, making them resistant to the available antibiotics in use (Fauci and Marston, 2014). In a recent joint statement, the Centers of Disease Control and Prevention, together with a number of national health organizations, succinctly highlighted the dilemma that is inherent in any antimicrobial usage policy: ‘The more we use antibiotics, the more we contribute to the pool of antibiotic-resistant microbes. The development of resistance is an inevitable by-product of exposure to antibiotics. All antibiotic use, whether warranted or not, places selection pressure on bacteria, and some organisms that possess genetic mutations will survive antibiotic treatment’ (CDDEP, 2012: 1). The slowly emerging aspect of AMR emphasizes that the antecedent conditions that could lead to a full-fledged disaster are always present, and that if we reach the post-antibiotic era it is unlikely that we will return to the normalcy we enjoy today. In this sense, the problem of AMR will be one of constant vigilance—trying to keep the slowly emerging aspects of the disaster at bay.

While the notion of a slowly emerging disaster itself is not without its problems, it is still *prima facie* plausible to conceptualize it as a possible type of disaster. Further, we may still have good policy-based reasons for approaching the issue of AMR as a slowly emerging disaster.

## AMR as a Slowly Emerging Disaster

So far, we have discussed the conceptual plausibility of describing AMR as a slowly emerging disaster. It will now be important to consider if and why such a conceptualization would be useful for the identification of suitable solutions to the problem. In this article, we wish to argue that understanding AMR as a slowly emerging disaster, rather than an acute medical problem, can account more comprehensively for the scale of the challenge and its societal implications.^10^ More specifically, we suggest that framing the policy response to AMR as a slow emerging disaster provides five advantages.

### Emphasis on Mitigation

Understanding AMR as a disaster should lead us to consider solutions to the problem that are appropriate for other kinds of disaster. In the field of disaster management, a strong emphasis is placed on the development of solutions that can help to mitigate through risk reduction strategies, rather than entirely avoid the consequences of a disaster (Wisner *et al.*, 2011; Smith, 2013). Similar strategies are increasingly gaining recognition in the prevention of (re-) emerging infectious diseases (Heymann and Dar, 2014).^11^ Given that there is a current lack of development of new antibiotics, mitigation strategies to reduce transmission of infections and prolong the effectiveness of antibiotics (e.g., by rationing their use) could complement efforts to boost research into new antibiotics. This would not constitute a replacement of existing strategies to develop new drugs and technologies to counter AMR, but rather an acknowledgement that, *ceteris paribus*, existing strategies fail to halt or reverse the trend towards more extensive drug resistance. In addition, understanding AMR as a predictable and unavoidable slowly emerging disaster—for which we can only mitigate consequences, rather than eliminate its causes—should lead us to rethink the often implausible rhetoric of combating and eradicating infectious diseases. However, mitigation strategies are less likely to succeed in those regions of the world that suffer from a high burden of infectious diseases and where the availability of effective antibiotics is consequently of particular importance (Selgelid, 2007; Laxminarayan and Heymann, 2012).

### International Cooperation and Communication

Existing policies on AMR are usually restricted to a national or regional context, and to very specific groups of stakeholders. Examples of such policies include regulations and guidelines for medical or veterinarian use of antibiotics, or initiatives to promote more conservative or ‘prudent’ drug prescription. However, given AMR’s global impact, which cannot be managed within the confines of national borders, broader efforts at the international level to address the challenge are necessary (Laximinarayan *et al.*, 2013). Existing strategies to deal with international disasters could offer a valuable source of information and infrastructure for improving dialogue and cooperation among international stakeholders to develop an integrated response to AMR (Thomalla *et al.*, 2006). This includes, for instance, the need for global surveillance to keep track of the development of resistance and to develop responses based on epidemiological evidence (WHO, 2014). However, there is also a clear need to share and transfer resources to those areas that are particularly affected by AMR, but may lack the means to appropriately address the problem themselves. Since many less-developed countries are particularly affected by the consequences of AMR, a transfer of resources and know-how will be a crucial aspect of a successful response to AMR. This also applies to the transfer of strategies to communicate and manage risks to the general public, as campaigns to raise the general awareness of AMR and its causes remain low (McNulty *et al.*, 2007, 2010). Risk communication systems for disasters are generally more widely established and offer tool kits for responders and policy makers to explain complicated or unpopular decisions.^12^ Such tool kits may be a useful addition to ongoing efforts to educate the public about risks of AMR—though they will need to be suitably altered to adapt to the situational features of a slowly emerging disaster that differ from the kinds of paradigmatic disaster situations for which these cooperation and communication strategies were designed.

Understanding AMR as a slowly emerging disaster may also help to conceptualize the problem in a way that is more accessible to the general public—a stronger focus on the inevitability of its occurrence, and an emphasis of the fact that AMR can affect anyone could be useful to engage the general public to a greater extent than is currently the case.

### Policy Focus on Future Resilience

Understanding AMR as a slowly emerging disaster would underline the need for future policies that establish resilience to the threat that drug resistance poses. Resilience is a critical concept in disaster management and aims at the creation of governance structures following a disastrous event that can better address and withstand comparable events in the future (Adger *et al.*, 2005; National Research Council, 2012). A focus on resilience accepts that the occurrence of disastrous events cannot itself be controlled, but that their effect depends on the organization, preparedness and response of society. A stronger focus on resilience in the case of AMR would help to widen the scope of policy focus beyond emphasizing the need for new therapeutic agents to strategies, which reduce the dependency on antibiotics. This could, for example, be achieved by better infection control and greater public awareness for appropriate use of antibiotics and ways to avoid infection, e.g., in the case of sexually transmitted drug-resistant infections, which already pose a major health threat in some countries (CDC, 2013). However, resilience will also depend on the ability to provide safe, high-quality health care with active infection management. Strengthening institutions in less-developed countries would help to reduce the risk of nosocomial infections and reduce AMR-related health inequities between wealthier and poorer nations.

### Recognizing the Interdisciplinary Nature of the Challenge

AMR is a multidimensional health challenge that has been described as ‘the perfect storm in public health’ (Gould, 2009). While the emergence of AMR itself is a biomedical process, its causes are incredibly complex and encompass a wide range of social and policy dimensions. These include, but are not limited to, the use of antibiotics in agriculture, access to health care resources in less-developed countries, health literacy and adherence to treatment, economic considerations of cost-effective treatments and psychological insights into antibiotic prescribing and use (e.g., Levy, 2002; Levy and Marshall, 2004; Grigoryan *et al.*, 2007; Anomaly, 2009; Smith and Coast, 2013). Understanding AMR as a slowly emerging disaster with not only a large number of contributing causal factors but also immense societal impact on a wide range of health care services and social interactions highlights both the need to act and the comprehensiveness of any solution with a chance of being successful. Tackling AMR will require the collaboration of a large number of national and international actors from different disciplinary and professional backgrounds, as well as the use of considerable resources. Learning from collaboration in previous disasters could offer a basic blueprint for action and serve as an example of successful response to the problem of AMR. There is an increasing number of international networks to develop interdisciplinary responses and prevention campaigns to the threat of (re-)emerging infectious diseases, yet their success in less-developed regions is often hindered by the lack of financial means (Heymann and Dar, 2014).

### Emphasis on Risk Sharing

As with disasters that make everyone more vulnerable, requiring collective action to successfully bring the disaster to an end, the focus on risk sharing makes our response to AMR take into account that policy and action undertaken needs to fairly balance both the benefits and burdens associated with trying to mitigate AMR. The focus on risk sharing not only emphasizes that every part of society—government, citizenry and private sector—needs to be active in taking steps to reduce risk, but it also brings moral considerations to the forefront in our response. A focus on risk sharing will rely on values such as stewardship, solidarity and sustainability underpinning which responses we should think are appropriate to deal with AMR. Moreover, placing an emphasis on risk sharing also allows us to identify and respond to those individuals and institutions responsible for contributing to the risk associated with AMR that we share. The antecedent conditions of a slowly emerging disaster not only allow us to identify those factors that are causally responsible for the development of the problem, but also help to trace whom and to what extent we should hold people morally responsible for their causal contribution to the problem.^13^

## Conclusion

In this article we have argued that, given current thinking about disasters and ways in which we might frame policy responses to public health issues, it can be plausible for AMR to be described as a slowly emerging disaster. In particular, we have suggested that framing the problem of AMR in these terms may be beneficial for at least five reasons. There are also legitimate concerns, however, over the use of overly negative or alarming terminology of disaster in relation to public health problems.

The first, as alluded to earlier, is the proliferation of disaster language. There is a serious concern about inducing a kind of ‘disaster fatigue’ among professionals and the public if we attach the label of disaster on to too many problems. For instance, in preparing for the predicted H5N1 pandemic, only for the less virulent H1N1 pandemic to have arrived first, many people expressed skepticism about the anticipated disastrous consequences of a pandemic that did not materialize. The more public health emergencies or disasters that are declared, the less power such a designation has to motivate individuals and institutions to action (Selgelid and Enemark, 2008). Due consideration must be taken in applying the label of disaster to public health problems, even the label of slowly emerging disasters.

Second, the use of disaster language or metaphors might also cause apprehension in terms of the associations it hearkens in how we have responded in the past to public health threats. Talk of disasters—especially ones like AMR that threaten the progress of modern medicine—might evoke ideas of sanatoriums or leper colonies and the use of severe restrictive measures, such as quarantine and isolation. The prime worry being that in responding to infectious disease threats in the past, the use of such metaphors have been used to make it easier to, according to Ross (1986: 18), ‘sacrifice people and their rights’. They may also lead to the proposal of solutions to problems that have been described as similar. AMR, for instance, has been described as akin to global warming, arguably another type of slowly emerging disaster (Laxminarayan and Brown, 2001; Herrman and Laximinarayan, 2010). However, while there are important similarities between these two challenges, they also differ in significant respects (Littmann, 2014). Using terminology that compares highly complex problems, which display unique and distinctive characteristics, could obfuscate these differences.

A third worry about the use of disaster terminology, voiced by Nerlich and James (2009), is that rather than serving as a call to action, the use of apocalyptic scenarios may unintentionally have the opposite effect; this is to say that the public may respond to the announcement of an impending and unavoidable disaster with fatalism. They may simply conclude that any action will come too late and be ineffective anyway, so there is no point in changing their behaviour or practices. Since the current scale of AMR is a human-caused problem and its further spread can be influenced by human action, it would be counterproductive to conjure up an image that suggests the impending disaster is inevitable. A balance needs to be struck between motivating action and inducing feelings of futility or paralysis.

A fourth worry is that a disaster label or frame can be (mis)used for the putative normative implications disasters are thought to bring about. In particular, there is a strong presumption that what may be immoral or illegal during times of normalcy could be rendered permissible during times of disaster.^14^ It is possible that a disaster labeling or framing could be adopted to seek an alteration in how the moral standards that govern how we should respond to AMR are weakened to make our efforts easier—think here, for instance, of the limitation and derogation of rights during times of disaster (United Nations, 1985). This raises both conceptual and ethical issues that need to be further considered in how we apply the label of disaster to different circumstances.

Concerns such as these should be taken seriously and weighed against the potential benefits of mobilizing a public response to AMR by describing it as a slowly emerging disaster. However, even if the concept of AMR as a slowly emerging disaster is found to be unsuitable for wider public communication, we believe that it may still carry weight for policy makers and health care professionals. For these groups, the classification of AMR as a slowly emerging disaster may serve less as a call to action and more as an opportunity to apply and transfer experiences collected during previous disasters, which approximate the scale and complexity of the challenge that AMR presents. And while AMR undoubtedly presents unique and novel challenges, components of a comprehensive response will be comparable to the reaction to other public health disasters. Framing AMR as a slowly emerging disaster underlines this point and would help us to build a response on the knowledge, practices and infrastructure that have proved to be successful in the past.

## Notes

1. There are different microorganisms that can become resistant to therapeutic and preventative measures, including bacteria, fungi, viruses and parasites. AMR can include any one of these microorganisms. In the course of our article, however, we shall focus on antibiotic resistance. Nevertheless, our arguments can also apply to other areas of AMR.

2. The UK Prime Minister also recently commissioned a report on AMR that called it a ‘global health crisis’ (Review on Antimicrobial Resistance, 2015).

3. Within the literature and public policy documents, the terms ‘disaster’ and ‘emergency’ are often used interchangeably—along with other terms, such as crisis, catastrophe, calamity, tragedy or hazard. While some people distinguish these terms to refer to different kinds of circumstances, for our present purposes nothing much hangs on which term is selected. Further, since people have tended to refer to ‘slowly emerging’, ‘slowly unfolding’ and ‘slowly evolving’ disasters, we shall primarily speak about ‘disasters’ in this article.

4. It also should be noted here that while we speak of disasters as events, this is not to presuppose or rule out the possibility of conceiving of disasters as processes. Generally speaking, behaviorists view disasters as events caused by natural hazards. Disasters *qua* events are departures from the social and cultural practices that constitute normalcy. Structuralists view disasters as functions of ongoing social, historical and/or political processes. On this view, disasters do not just happen; they result within a specific set of human-environment relations. See, for instance, Fritz (1961), Quarantelli (1998), Bolin and Stanford (1998).

5. For an account of other central characteristics of disasters, see Viens (2012).

6. See, e.g., the US National Institutes of Health definition: http://www.niaid.nih.gov/topics/tuberculosis/understanding/whatistb/pages/tbdefinitions.aspx [accessed 1 May 2015].

7. We thank an anonymous referee for pressing this point.

8. That is to say, it provides the conceptual space for thinking that it is one type of disaster that comports with our ordinary thinking about disasters and yet also comports with the problem of AMR. Of course, if one believes that temporal boundaries should not be a criterion of disasters, then that provides even less of an obstacle to thinking that slowly emerging disasters might be plausible. We have chosen to examine the idea of a slowly emerging disaster as being consistent with a temporal boundary criterion for disasters because (i) it is a feature that is implicitly and explicitly found in many conceptions of disaster and (ii) there are good reasons for thinking that a temporal boundary criterion should be part of our conceptualization of disasters.

9. Many anthropogenic public health threats, such as climate change and water pollution, also share this slowly emerging aspect, though disagreement still exists as to whether they are disasters or disasters in the making as a result. These threats also include apparent natural disasters, such as famine and drought, which often have anthropogenic (most often political and economic) causes. In such cases, we often do not include the antecedent conditions as part of the disaster event itself—e.g., the political corruption and malfeasance that leads to depriving a community of means to produce or access sufficient levels of food. According to structuralists, however, if we understand disasters as processes and not events, we can better account for the relationship between what we have called the antecedent conditions and the disaster itself. Nevertheless, the idea of a slowly emerging disaster is not meant to presuppose a structuralist view of disasters.

10. This, of course, does not preclude that there may be different ways to frame the problem of AMR. For instance, AMR has also been framed as a problem of global health security (Buckland-Merrett, 2013) or a problem of global public goods (Höjgård and Vågsholm, 2014). We do not think that these framings have to be seen as alternative or competing framings, necessarily. It is useful, however, to isolate particular framings to explore their advantages and disadvantages. Successful responses to AMR should seek to borrow what is useful from whichever framings shed light on the issue or intervention at hand.

11. One anonymous reviewer raised the point that mitigation need not be the only perspective emphasized. Given the situational features of slowly emerging disasters, we may also have good reason for emphasizing adaptation—similar to some strategies for climate mitigation and adaptation. On this view, attempts to develop new therapies and technologies are a form of adaptation that recognizes and accepts that that drug resistance is inexorable.

12. See, for example, the UK guidelines for risk communication, available at: https://www.gov.uk/government/uploads/system/uploads/attachment_data/file/60907/communicating-risk-guidance.pdf [accessed 1 May 2015].

13. We explore this topic further in our article, ‘Individual and Institutional Responsibility for Antimicrobial Resistance’ (manuscript).

14. For more on this, see Viens (2012) and Viens and Selgelid (2012).
